# Genetic basis of cardiovascular aging is at the core of human longevity

**DOI:** 10.20517/jca.2022.06

**Published:** 2022-04-14

**Authors:** Ali J. Marian

**Affiliations:** Center for Cardiovascular Genetics, Institute of Molecular Medicine and Department of Medicine, University of Texas Health Sciences Center at Houston, Houston, TX 77030, USA.

**Keywords:** Longevity, life span, health span, genetics, progeria, APOE4, FOXO3, mTOR, DNA damage

## Abstract

Aging is an archetypical complex process influenced by genetic and environmental factors. Genetic variants impart a gradient of effect sizes, albeit the effect sizes seem to be skewed toward those with small effect sizes. On one end of the spectrum are the rare monogenic premature aging syndromes, such as Hutchinson Gilford Progeria Syndrome, whereby single nucleotide changes lead to rapidly progressive premature aging. On the end of the spectrum is the complex, slowly progressive process of living to an arbitrary-defined old age, i.e., longevity. Whereas the genetic basis of rare premature aging syndromes has been elucidated, only a small fraction of the genetic determinants of longevity and life span, time from birth to death, have been identified. The latter point to the complexity of the process and involvement of myriad of genetic and non-genetic factors and hence, the diluted effect of each determinant on longevity. The genetic discoveries point to the involvement of the DNA damage and activation of the DNA damage response pathway, particularly in the premature aging syndromes. Likewise, the insulin/insulin-like growth factor 1/mTOR/FOXO pathways have emerged as major regulators of life span. A notable fraction of the genetic variants that are associated with life span is also associated with age-related cardiovascular diseases, such as coronary artery disease and dyslipidemia, which places cardiovascular aging at the core of human life span. The clinical impact of the discoveries pertains to the identification of the pathways that are involved in life span, which might serve as targets of interventions to prevent, slow, and even possibly reverse aging.

## INTRODUCTION

Aging, defined as a gradual decline of physiological and biological functions of cells, organs, and organisms, is a quintessential complex phenotype. At the clinical level, aging is associated with several diseases, such as coronary artery disease (CAD), osteoarthritis, diabetes mellitus, type II diabetes, and cancer, to name a few. At the molecular and cellular levels, aging is associated with genomic instability, telomere attrition, epigenetic alterations, impaired proteostasis, dysregulated nutrient sensing, mitochondrial dysfunction, cell senescence, stem cell exhaustion, and altered cell-cell communications [Table 1]^[1]^. These changes are referred to as the biological hallmarks of aging^[1]^.

There is considerable inter-individual variability in clinical aging and its biological hallmarks, as well as in the susceptibility to age-related disorders. The variability is due to differences in the genetic and environmental factors, which have been partially delineated. To illustrate the influence of the genetic factors in aging, Sir Winston Churchill and Sir Ernest Henry Shackleton are epitomized as the prototypic examples. Shackleton, the healthy Antarctica explorer, died at 47 years of age from an apparent heart attack on his fourth expedition to the South Pole, whereas Churchill, the British statesman who was overweight and smoked 8 to 10 cigars a day, lived to the age of 90 years and died from a stroke.

Whereas longevity is defined as living to an arbitrary-define old age, life span is the maximum number of years one lives. A portion of the life span that is free of concomitant age-related diseases is referred to as the healthy life span or the health span. Contributions of the genetic factors to age-related diseases, such as atherosclerosis and CAD, as well as the responses of age-related diseases to preventive and therapeutic interventions are well recognized and partially elucidated^[2]^. However, genetic factors that determine life span and health span have been difficult to decipher for various technical reasons, including heterogeneity of the phenotype used in the genetic studies. Rare genetic disorders leading to premature aging syndromes provide insights into the mechanisms of aging at the molecular and cellular levels. Likewise, genome-wide association studies (GWAS) have been applied to identify the genomic loci that are associated with aging in the general and elderly populations. The purpose of this review is to discuss the genetic basis of human aging by focusing on two ends of the spectrum of aging, namely rare progeria and progeroid syndromes on one end and longevity on the other. The genetic basis of age-related disorders and likewise, the epigenetic basis of aging are not discussed. Likewise, the review article does not cover the genetic basis of aging in model organisms.

## HUMAN LIFE SPAN

Over the last century, the chronological age of the longest-living human has increased from about 110 years to 122 years^[3]^. The increase in the life span, time from birth to death, has raised the intriguing question of whether life span will continue to increase and whether there is a limit to the maximum life span of humans. Likewise, life expectancy, the average time one is expected to live, has progressively increased, and the median life expectancy has doubled over the last century. The increase in life expectancy, however, is largely due to the advances in health care, particularly the prevention and treatment of infectious diseases, agricultural revolution, and industrial developments. Life span, health span, and life expectancy are influenced both by genetic and environmental factors as well as their interactions. Likewise, these traits are influenced by age-related diseases, such as CAD and cancer. Nevertheless, there are complex intertwined and non-linear interactions between the environmental and genetic factors in determining life span, health span, and life expectancy. A notable example of the environmental factors that affect longevity is exposure to ultraviolet light, which is known to induce single- and double-stranded DNA breaks. The release of the double-stranded DNA breaks (DSBs) to the cytoplasm activates the DNA damage response (DDR) pathway, which induces inflammation and leads to cell senescence.

## GENETIC BASIS OF LIFE SPAN

The maxim by Johann Wolfgang von Goethe that “It’s in the anomalies that nature reveals its secrets” is nicely depicted in genetic disorders. Rare forms of genetic conditions provide an excellent opportunity to gain insights into the mechanisms that govern the pathogenesis of the common traits. In the case of aging, the phenotypic extremes of longevity provide excellent windows of opportunities to elucidate the genetic basis of aging, as they are less affected by the confounding effects of concomitant age-related phenotypes. On one end of the spectrum are individuals with extreme longevity, such as Jeanne Calment (1875–1997) of France, who was the longest living person. She lived 122 years and 164 days, despite smoking and drinking. On the opposite end of the spectrum are those with the monogenic disorders of premature aging, such as the Hutchinson Gilford Progeria Syndrome (HGPS), who typically die in the second decade of life.

### Genetic basis of premature aging syndromes

Premature aging involving multiple organs pertains to a heterogeneous group of genetic disorders with variable phenotypic expression. Given the focus of this review on the cardiovascular component of longevity, only selected progeria syndromes whereby cardiovascular involvement is prominent are discussed.

#### HGPS:

The syndrome, first described by Sir Jonathan Hutchinson and Hastings Gilford, is an exceeding rare premature aging condition that manifests during early childhood with a failure to thrive and features of aging, such as progressive alopecia, hearing loss, sclerotic skin, lipodystrophy, osteoporosis, and joint abnormalities, and diabetes mellitus^[4,5]^. Premature atherosclerosis is common and the leading cause of death because of myocardial infarction and stroke. The affected individuals typically die in the second decade of life because of progressive heart failure^[4]^.

The classic HGPS is caused by a single-base substitution within exon 11 of the *LMNA* gene, which encodes the lamin A/C (LMNA) protein^[6]^. The LMNA protein is a nuclear inner membrane protein with a diverse array of functions, which mainly pertain to the mechanical stability of the nuclear member, mechanosensing, chromatin organization, gene expression, and intracellular signaling^[7]^. The *LMNA* gene encodes two main and two minor isoforms. The A isoform (Lamin A) is a 664 amino acids (aa) protein, whereas the C isoform (lamin C) is shorter (572 aa) because it is the product of an alternative splice site (C>T transition) in exon 10 of the *LMNA* gene^[8]^. The C>T transition inserts six unique amino acids after codon 566 and induces premature termination of the protein. A third isoform, referred to as lamin C2, results from an alternative initiation site (ATG) in intron 1 of the *LMNA* gene, whereas the lamin Δ10 isoform is the consequence of skipping the entire 90 bases of exon 10, also by alternative splicing^[9]^. The lamin Δ10 is ubiquitously expressed, but the lamin C2 is uniquely expressed in the germline cells^[10]^.

**Figure 1.**
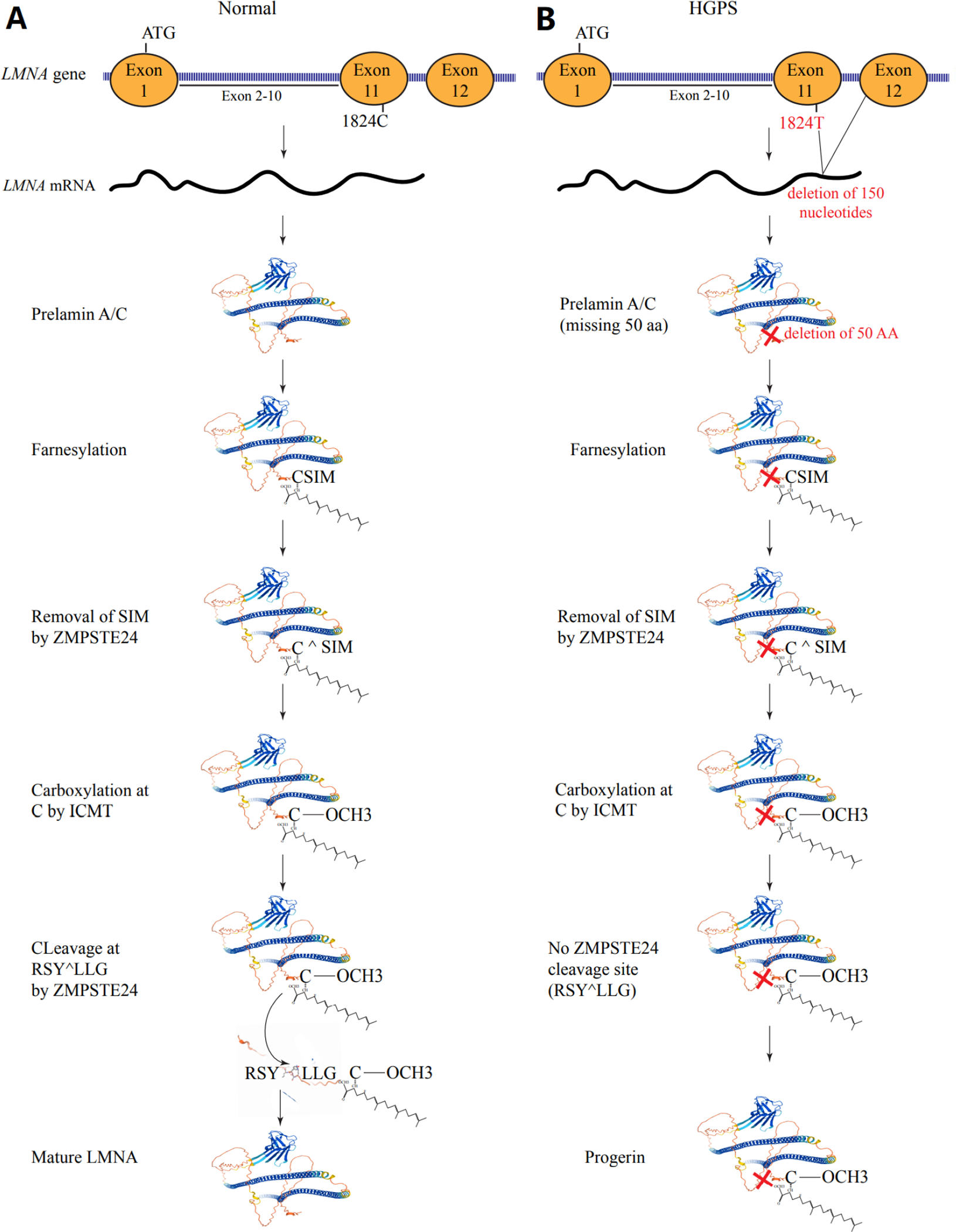
Post-translation modification of LMNA. (A) The *LMNA* gene, which is comprised of 12 exons, encodes the prelamin A/C protein, which undergoes farnesylation of the cysteine residue at the CaaX motif at the COOH terminus (amino acids CSIM), which is then cleaved by ZMPSTE24 or FACE1, leading to the removal of the SIM amino acids. This is followed by carboxylation of the cysteine residue by isoprenylcysteine carboxyl methyltransferase (ICMT). The carboxylated protein is cleaved at RSŶLLG recognition motif by the ZMPSTE24, resulting in the removal of the last 18 amino acids from the protein and producing the mature LMNA protein. (B) In the classic Hutchinson-Gilford Progeria Syndrome (HGPS), a C>T transition in exon 11 of the gene, while a synonymous variant, introduced a cryptic splicing site, which removes 150 nucleotides from the mRNA. Thus, the prelamin A/C protein has a deletion of 50 amino acids near the COOH terminal of the protein. The protein undergoes farnesylation, removal of the SIM amino acids at the COOH terminal, and carboxylation of the cysteine residue. However, because of the deletion of the 50 amino acids, the ZMPSTE24 recognition site is deleted, and consequently, the farnesylated/carboxylated mutant pre-LMNA referred to as progerin, accumulates in the nucleus.

The LMNA protein is expressed as prelamin and undergoes extensive post-translational modifications to generate the mature LMNA. Initially, a cysteine residue in the cysteine-serine-isoleucine-methionine motif at the carboxy-terminal domain is farnesylated by a farnesyltransferase. The farnesylation is followed by cleavage of the motif by zinc metalloprotease CAAX prenyl proteases 1 (FACE1) or zinc metallopeptidase STE24 (ZMPSTE24), which leaves behind the farnesylated cysteine. The latter undergoes carboxymethylation by isoprenylcysteine carboxyl methyltransferase, which is followed by cleavage of the last 15 aa from the protein by the ZMPSTE24 protease and generation of the mature LMNA protein [Figure 1].

The causal mutation in HGPS is a C>T transition, which changes the codon 608 from GGC to GGT^[6]^. The mutation is synonymous, as both codons define glycine (p.Gly608Gly). The nucleotide change, however, activates a cryptic splice site, which induces an in-frame deletion of the last 150 bases of exon 11, encoding 50 amino acids^[6]^. The deletion of the 50 amino acids affects the maturation of the LMNA protein, including cleavage of the prelamin A by the FACE2 or ZMPTSTE24 protease after post-translational farnesylation and carboxylation. Consequently, a new protein that lacks the last 50 aa of the full-length LMNA is synthesized, which is referred to as progerin [Figure 1].

The role of the *LMNA* gene in premature aging syndromes is further supported by the identification of additional rare pathogenic variants (PVs) in patients with HGPS and other forms of progeroid syndromes^[11,12]^. A notable example is the identification of the p.D300N (p.Asp300Asn) mutation in the *LMNA* gene in cases with the progeroid syndrome, including a patient in whom the cardiovascular system was predominant if not the exclusive organ involved^[12,13]^. The phenotype in the latter case was characterized by progressive premature coronary atherosclerosis, conduction defects, atrial fibrillation, calcified valvular heart disease, and restrictive cardiomyopathy, and was referred to as the “non-syndromic cardiac progeria”^[12]^.

The discovery of the *LMNA* gene as a cause of HGPS and progeroid syndromes raised interest in the role of the LMNA protein in aging in the general population. Several findings have supported the involvement of the LMNA proteins in aging, including evidence of an age-dependent decline in the ZMPTSTE24 expression in response to oxidative stress and a gradual accumulation of prelamin A in various cells, including vascular smooth muscle cells^[14]^. Likewise, aberrant splicing of the LMNA leading to accumulation of progerin is implicated in aging^[15]^. Despite these observations, the role of the LMNA in physiological aging remains largely unsettled.

Several mechanisms are implicated in the pathogenesis of HGPS, which also might have implications for aging in general. Accumulation of the farnesylated progerin in the inner nuclear membrane and the loss of nuclear membrane mechanical integrity are among the major mechanisms involved in the pathogenesis of progeroid syndromes^[16,17]^. The salubrious effects of treatment with farnesyltransferase inhibitors provide credence to the pathogenic effects of the farnesylated progerin in HGPS^[18]^. However, the beneficial effects are only partial, which indicates the multifarity of the pathways involved in the pathogenesis of HGPS.

The LMNA protein interacts with chromatin through the lamin-associated domains^[19]^. LMNA also recruits the histone-modifying enzymes, such as lysine demethylase 5 A and B (KDM5A/B), histone-lysine N-methyltransferase (SUV39H1), and histone deacetylase 3 (HDAC3) to chromatin^[20–22]^. In addition, LMNA regulates methylation and acetylation of the histone proteins and the CpG sites^[19,23–25]^. These epigenetic changes affect gene expression. Therefore, not surprisingly, epigenetic remodeling of chromatin has been implicated in the pathogenesis of progeroid syndromes.

**Figure 2.**
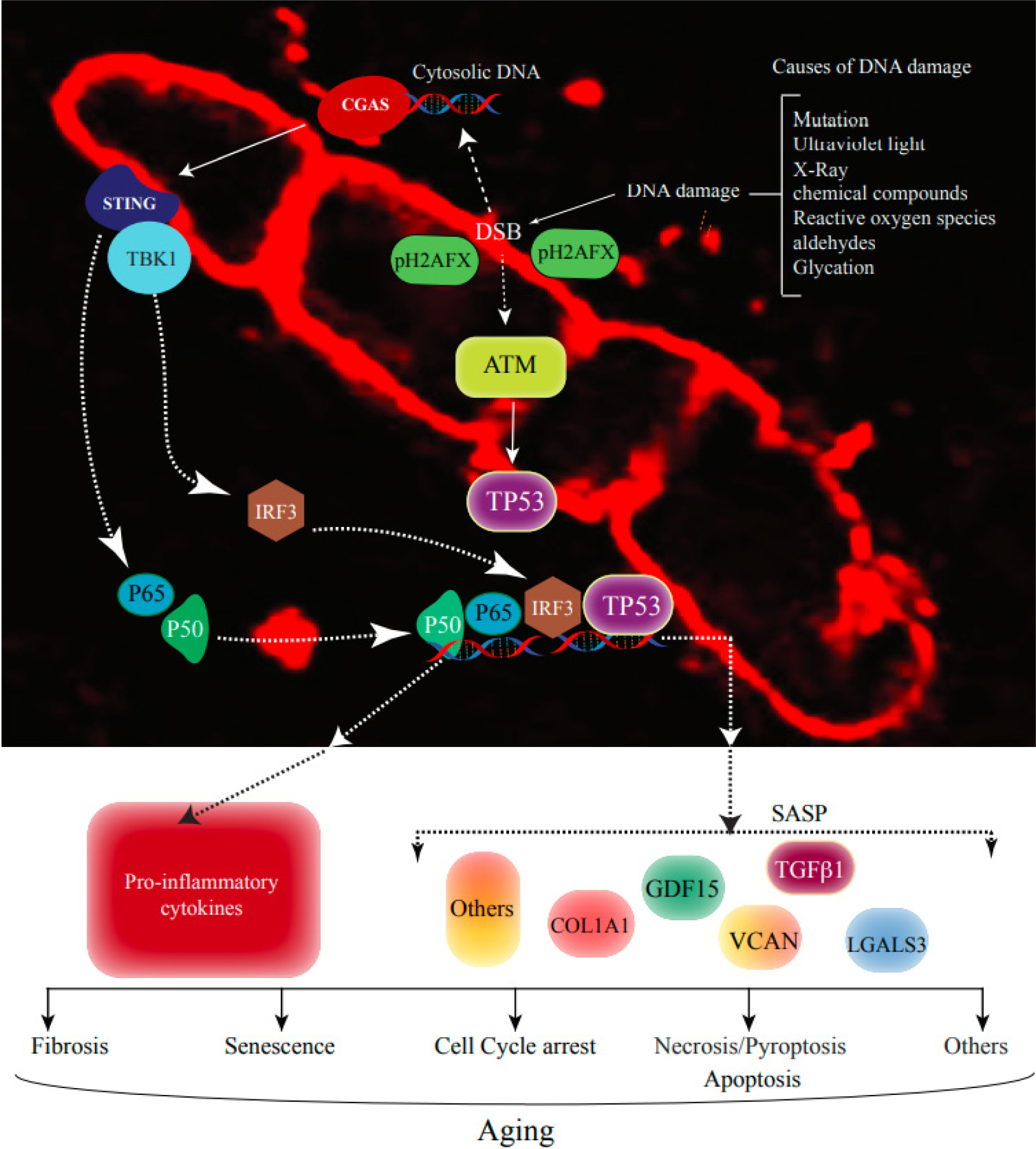
DNA damage response in aging. The genome is not only affected by the mutations but also by various endogenous, such as reactive oxygen species, and exogenous, such as ultraviolet light, resulting in various forms of DNA damage, most notably double-stranded DNA breaks (DSBs). The red color staining depicts the cardia myocyte LMNA protein, which resides in the inner nuclear membrane close to the chromatin. LMNA is involved in induction as well as repair of the DSBs and has a crucial role in nuclear membrane integrity. In the presence of LMNA mutations or deficiency, DSBs are increased and released into the cytoplasm, which is then sensed by the cyclic GMP-AMP synthase (CGAS) followed by activation of stimulator of interferon genes protein 1 (STING1) and TANK binding kinase 1 (TBK1). STING1 activates the nuclear factor kappa B (NFκB) components p65 and p50, whereas TBK1 phosphorylates interferon regulatory factor 3 (IFR3) which translocates into the nucleus and induces the expression of proinflammatory genes. Several proteins are recruited to the site of the DSBs, including the ataxia-telangiectasia mutated (ATM), which phosphorylates H2 histone family member X (H2AFX) and the tumor suppressor protein 53 (TP53). Activated TP53 translocates into the nucleus and induces the expression of genes involved in senescence-associated secretory phenotype (SASP), which collectively mediates molecular and cellular phenotypes of aging such as cell cycle arrest, senescence, fibrosis, apoptosis, and others.

LMNA is also involved in genomic stability through interactions with several proteins involved in the actively transcribed chromatin domains, formation of chromatin loops, and processing of topoisomerase and related enzymes^[26,27]^. Moreover, progerin stalls the DNA replication fork by recruiting components of the replisome away from the repair sites and increasing the repair via error-prone nonhomologous end-joining (NHEJ)^[26,28–30]^. A consequence of genomic instability is increased DNA damage, particularly DSBs. Increased DSBs, along with the loss of nuclear membrane integrity, lead to the release of the DSBs, upon rupture of the nuclear membrane, into the cytoplasm and activation of the DDR pathway^[31–33]^. The cytoplasmic DNA is sensed by the DNA sensors, such as CGAS (a.k.a. cGAS), ZBP1, and AIM2. The CGAS protein, when binding to the DNA, activates the STING1/TBK1 kinase pathways, which target transcriptional factors nuclear factor kappa B (NFκB) and interferon regulatory factor 3 (IRF3) for activation. Consequently, expression of the pro-inflammatory cytokines is induced, and cell senescence is instigated [Figure 2]. In addition to activation of the pro-inflammatory pathways through the NFκB and IRF3 transcription factors, increased DSBs activate the ATM/CHK2/TP53 pathway, which leads to the expression of genes involved in growth arrest and senescence^[15,32,34–36]^.

The LMNA protein is also the link between the mechanical forces originating at the extracellular matrix and the chromatin. Consequently, dysregulation of mechano-sensing and mechano-transduction, leading to activation of several signaling pathways, including the mitogen-activated protein kinase (MAPK) pathway, is implicated in the pathogenesis of progeroid syndromes^[37]^. The external mechanical forces are initially sensed by the integrins and transmitted through the intermediary filaments, such as actin and desmin, to the linker of nucleoskeleton and cytoskeleton (LINC) complex^[38]^. The LINC complex, comprised of SUN and nesprin proteins, binds to the LMNA protein, which directly interacts with several chromatin proteins. Through this chain of interactions, LMNA regulates signaling pathways and the response of the chromatin and gene expression to external stimuli.

Dysregulated mechanistic target of rapamycin (mTOR) and insulin-like growth factor 1 (IGF1) signaling, impaired nutrient sensing, and autophagy are implicated in the pathogenesis of progeroid syndromes, laminopathies, as well as aging in the general population. Genetic and pharmacological inhibition of the mTOR signaling pathways, as well as intermittent fasting and restriction of branched-chain amino acids, are shown to increase life span, including in a mouse model of progeria, and reduce age-related diseases^[39–43]^.

Telomere shortening, which is an essential component of aging, is also implicated in the pathogenesis of HGPS^[44]^. LMNA interacts with the telomere repeat-binding factor 2 (TRF2) protein, which is a core component of the shelterin complex^[45,46]^. This interaction is essential for the formation of the telomere loops and telomere stability. However, in contrast to LMNA, progerin does not interact with the TRF2^[46]^. The loss of the interaction of LMNA with TRF2 leads to telomere instability and attrition^[46]^. In accord with these mechanistic data, the average telomere length is markedly reduced in patients with HGPS as well as in the LMNA-deficient cells^[44,47]^. Length-independent telomere dysfunction also leads to cardiac myocyte senescence^[48]^. The role of the LMNA in this process, however, remains unclear.

Several additional mechanisms, including mitochondrial dysfunction and increased oxidative stress, are also implicated in the pathogenesis of HGPS, which are discussed elsewhere^[17]^.

The phenotypic spectrum of mutations in the *LMNA* gene includes a diverse array of diseases, involving multiple cell types and organs, which are collectively referred to as laminopathies^[49]^. The classic laminopathies, in addition to the premature aging syndromes, include cardiomyopathies, muscular dystrophies, lipodystrophies, restrictive dermopathy, and a few others^[50]^. Cardiac involvement in laminopathies is notable and manifests as dilated cardiomyopathy, conduction defects, and arrhythmias^[51]^. Cardiac disease is a major cause of mortality and morbidity in laminopathies^[51]^.

It is intriguing to postulate the presence of shared mechanisms between HGPS or the progeroid syndromes and other forms of laminopathies, including cardiomyopathies. A shared mechanism(s) might also be operant, as one allele of the *LMNA* gene undergoes alternative splicing in the HGPS and does not express the mature LMNA, but the second allele expresses the wild-type protein. Consequently, in addition to an expression of progerin, accumulation of the farnesylated progerin, there is also a partial deficiency of the wild-type LMNA protein in HGPS. The latter mechanism is shared with other forms of laminopathies caused by the haploinsufficiency mechanism. In support of this notion, increased DSBs and activation of the DDR pathways are common pathogenic mechanisms in laminopathies as well as in progeroid syndromes[19,32].

#### Werner syndrome:

Werner syndrome is a rare premature aging syndrome, with the clinical manifestations largely similar to those of HGPS, except that it is relatively a milder form and starts during adolescence and later. The clinical manifestations of the syndrome, first described by Otto Werner in 1904, includes tardy growth spurt during adolescence, short stature, graying and loss of hair, atrophy of skeletal muscles, subcutaneous fat, premature cataract, diabetes, and atherosclerosis as well as increased risk of cancer (Reviewed in Ref.^[52]^).

Werner syndrome is caused by compound loss-of-function mutations in the *WRN* gene, which encodes RecQ-type DNA helicase^[53]^. The protein has exonuclease activity and is rapidly recruited to the sites of DSBs for repair. WRN is involved in NHEJ and homologous recombination through interactions with several DNA damage and repair proteins, including ATR, ATM, H2AFX, XRCC4, XRCC5 (Ku80), XRCC6 (Ku70), and DNA-dependent protein kinase (PRKDC or DNA-PK) among others^[54,55]^. In addition, the WRN protein is involved in epigenetic regulation of chromatin structure and function, telomere attrition, cell senescence, and autophagy^[56,57]^. These findings point to the involvement of various hallmarks of aging in patients with Werner syndrome, as in the case of HGPS, and identify genome instability as a core mechanism in the pathogenesis of progeria and progeroid syndromes.

### Genetic basis of life span as a complex trait

Each human genome contains over 4 million DNA sequence variants as compared to the reference genome, the vast majority of which are single nucleotide variants (SNVs) or polymorphisms (SNPs) (Reviewed in Ref.^[58]^). The genetic variants include several thousand functional variants and several hundred PVs or likely pathogenic variants^[58]^. Functional variants are expected to impart biological effects and influence the expression of the cellular and clinical phenotype by affecting the structure and functions of their respective proteins. Aging, life span, and health span are no exception and are influenced by genetic factors.

The commonly used approach to genetic studies of complex traits is the GWAS, whereby the population frequencies of the genetic variants, typically > 500,000 variants, are compared between thousands of cases and controls. In the case of genetics of life span, a proper design is challenging for various reasons, including the obvious fact that age at the time of the enrollment in the GWAS does not capture the life span of the participants. Likewise, retrospective genetic studies of life span have to consider the confounding effects of differences between the generations. An alternative approach to genetic studies of life span is using the life span of the parents and imputing their genotypes from the genotypes of their offspring. Using longevity, or an arbitrary-defined age, as the phenotype of interest requires proper matching of the cases and the controls in order to reduce the confounding effects of age-related diseases. Nevertheless, despite these challenges, the GWAS have led to the identification of several loci/genes that are associated with human longevity, parental life span, and health span, which are briefly discussed.

#### CDKN2B-AS1:

The gene, also called *ANRIL*, is located at the *CDKN2A-CDKN2B* locus on chromosome 9p21 and transcribes a long non-coding RNA with multiple isoforms. The *CDKN2A* and *CDKN2B* genes code for the cyclin-dependent kinase inhibitors 2A and 2B, respectively, which are major cell cycle suppressors. *ANRIL* has several functions, including transcriptional silencing of the gene at the locus as well as distant genes, through recruitment of the polycomb repressive complex 1 and 2 (PRC1 and PRC2, respectively) to the loci^[59]^. Likewise, *ANRIL* is also implicated in the regulation of the expression of several miRNAs as well as acting as a sponge for miRNAs^[60]^.

*CDKN2B-AS* locus has been associated with longevity in independent populations^[61,62]^. The *CDKN2B-AS* or *ANRIL* regulates not only the expression of cell cycle regulator genes *CDKN2A* and *CDKN2B* but also the cellular response to interferon, which collectively leads to induction of cell cycle arrest, expression of senescence-associated secretory phenotype (SASP), and cell senescence^[63,64]^. The 9p21 was the first locus mapped as a susceptibility locus for CAD and subsequently several other traits, such as diabetes mellitus^[65–67]^. Given that this locus is strongly associated with several age-related phenotypes, the association of *CDKN2B-AS1* with longevity might be secondary to its well-established associations with CAD, metabolic syndromes, and cell senescence.

#### FOXO3:

The encoded protein, referred to as forkhead box protein O3 (FOXO3), is a member of the forkhead family of transcription factors. FOXO3 protein is involved in insulin and IGF-1 signaling, autophagy, mitochondrial function, inflammation, apoptosis, and others^[23,68,69]^. The IGF1 pathway was among the first implicated in longevity, as reduced expression of *daf2*, an insulin receptor-like gene, was shown to increase life span in *C. elegans*^[70]^. The insulin/IGF1 pathway plays an essential role in longevity in various species, ranging from *C. elegans* and *Drosophila* to mammalians^[71]^. Caloric restriction, which is the strongest intervention to increase longevity in various model organisms, functions through suppression of the insulin/IGF1 signaling pathway^[72]^. Accordingly, caloric restriction by attenuating the insulin/IGF1 signaling, suppresses the phosphoinositide-3-kinase (PI3K)/Protein Kinase-B (AKT) pathway, which is known to target FOXO3 for inactivation upon phosphorylation^[73]^. Through this mechanism, caloric restriction leads to activation of the FOXO3 transcription factor and induction of a large number of genes involved in various components of the hallmarks of aging.

Genetic variants in the *FOXO3* gene have been associated with longevity, a finding that has been replicated in several but not all studies^[61,74–77]^. The observed association of the genetic variants of the *FOXO3* gene with longevity is in accord with the well-established role of reduced insulin/IGF1 signaling in aging, as discussed above.

#### APOE:

The *APOE* gene codes for the apolipoprotein E (APOE), which is a major component of the chylomicron and is involved in lipid transport. The APOE protein has three common alleles referred to as E2 or ε2, E3 or ε3, and E4 or ε4. The alleles differ at amino acid positions 112 and 158. The E2 allele contains amino acid cysteine at positions and E4 arginine, but E3 has amino acids cysteine at position 112 and arginine at position 158. The APOE alleles have been associated with cardiovascular traits and dementia, with the E4 being the risk allele for higher levels of low-density lipoprotein cholesterol (LDL-C) and apolipoprotein B (APOB), and Lp(a) levels, as compared to the E2 alleles^[78–80]^. Clinically, the APOE4 variant is associated with an increased risk of CAD and Alzheimer’s disease^[78–80]^.

The *APOE* gene variants were among the first genes that were associated with longevity, a finding that has been replicated in several independent populations^[62,81,82]^. The E4 allele is more common in those with a shorter than those with a longer life span, i.e., the E4 allele is associated with lower odds of surviving to old age, whereas the E2 allele frequency is the opposite^[62,81,82]^. Given that the E2 and E4 alleles are associated with beneficial and deleterious cardiovascular effects and Alzheimer’s disease, respectively, the associations of these alleles with longevity are likely secondary^[78–80]^.

#### LPA:

The gene encodes apolipoprotein (a), which is a major constituent of lipoprotein (a). Plasma levels of lipoprotein (a), which is comprised of an LDL-C molecule attached to a glycoprotein apo(a) particle, exhibit large intra-individual variability, partly because of the presence of copy number variants in the *LPA* gene^[83,84]^. The copy number variants at the *LPA* locus are also associated with an increased risk of CAD, and valvular calcification and stenosis^[85–87]^. Genetic variants at the *LPA* locus are associated with parental life span as well as health span^[76,88]^. Association of the *LPA* locus with longevity might be secondary to its impact on age-related diseases.

#### LDLR:

The encoded protein is the low-density lipoprotein receptor (LDLR), which is the main cell surface receptor responsible for the uptake of the LDL-C. A genetic variant in the *LDLR* gene has been associated with paternal life span, likely through its influence on plasma LDL-C levels^[82]^.

#### LPL:

The encoded protein lipoprotein lipase is a triglyceride lipase on the vascular endothelial surface and is primarily responsible for the clearance of triglycerides from the blood^[89]^. A genetic variant in the *LPL* gene is associated with paternal life span^[82]^.

#### *CLESR2*/SORT1/*PSRC1* locus:

The locus on 1p13 has been associated with the plasma LDL-C levels and the risk of CAD in GWAS, and the *SORT1* gene has emerged as the most attractive candidate^[90]^. The SORT1 protein mediates protein transport from the Golgi apparatus to the lysosomes. An SNP at this locus (rs599839) is associated with paternal life span^[82]^.

#### *CHRNA3/5* locus:

The *CHRNA3* and *CHRNA5* genes encode the cholinergic receptor nicotinic alpha 3 and 5 subunits, respectively, which mediate fast signal transmission at synapses. Genetic variants near the *CHRNA3* and *CHRNA5* genes have been associated with parental life span, particularly in young and middle-aged men^[77]^. Age- and sex-dependent association of the *CHRNA*3/5 locus with longevity has been validated in a meta-analysis^[76]^. The effect on the life span might be secondary to disease-susceptibility because of the association of the variants at this locus with smoking, nicotine dependence, lung cancer, and peripheral arterial disease^[91]^.

#### ATXN2:

The gene codes for the ataxin-2 protein, which is a ubiquitously expressed gene involved in endocytosis, mTOR signaling, mitochondrial function, and ribosomal translation^[92]^. Expansion of the CAG repeats in the *ATXN2* gene causes Parkinson’s disease and spinocerebellar ataxia 2 (Reviewed in Ref.^[92]^). A SNP in the *ATXN2* gene has been associated with paternal life span in a meta-analysis of the GWAS in the UK biobank and Ancestry DNA populations^[82]^.

#### *MICA*/*MICB* locus:

The locus contains genes encoding major histocompatibility complex class I chain-related proteins A and B, which are highly polymorphic and involved in antigen presentation. The locus has been associated with the paternal life span^[82]^.

#### EPHX2/CLU:

The locus on chromosome 8 codes for bifunctional epoxide hydrolase 2 (EPHX2) and clusterin (CLU) as well as a microRNA. EPHX2 converts epoxides to dihydrodiols and is involved in fatty acid metabolism, whereas clusterin is a chaperone protein implicated in cell death and neurodegenerative diseases^[93,94]^. A SNP at this locus has been associated with paternal life span^[82]^.

#### SEMA6D:

Semaphorin-D, encoded by the *SEMA6D* gene, is involved in neuronal differentiation and development^[95]^. Meta-analysis of the Ancestry DNA and UK Biobank GWAS showed an association with an SNP in the *SEMA6D* gene and paternal life span^[82]^.

#### IP6K1:

The encoded protein Inositol hexakisphosphate kinase 1 (IP6K1) is a regulator of inositol synthesis, which is involved in signal transduction^[96]^. *IP6K1* is the only gene that is associated with maternal but not paternal life span^[82]^.

#### Missing longevity/life span/health span genes:

Whereas the role of the genetic variants in age-related traits, such as atherosclerosis, dementia, and osteoarthritis, has been partially elucidated, there are scant data on the genetic basis of longevity, life span and health span, independent of those related to the concomitant age-related diseases. Also noticeable is the paucity of the human molecular genetic variants that are associated with the hallmarks of aging, such as genomic instability, telomere attrition, and dysregulated nutrient sensing. The paucity contrasts with the complexity of the aging phenotype, which begets the involvement of a very large number of genetic variants in various hallmarks of aging. The paucity might be partly due to the challenges in identifying the culprit genetic variants, partly due to difficulties in designing proper studies and partly because of the small effect sizes of such genetic variants, which render them difficult to discern.

The paucity also raises the question about the overall contributions of the genetic variants to the aging phenotypes. The problem is further compounded, in contrast to several other traits, by the difficulties in the accurate estimation of the heritability of human life span or longevity. The latter is because of the presence of confounders, such as assortative mating, heterogeneity in the phenotypes assessed in the studies, and the effects of the environmental factors^[97]^. Overall, the estimates have varied from as low as less than 10% to as high as 48%^[98,99]^. Age-related phenotypes, however, have higher heritability, and in some instances, such as Alzheimer’s disease and a lesser extent coronary atherosclerosis, genetic factors are the main determinants^[100]^.

It is noteworthy that the GWAS of longevity and life span thus far have not identified genetic variants that influence functions of the mTOR pathway, except for those related to the *FOXO3* gene, as discussed earlier. This might reflect the complexity of the mTOR pathway, with the complex 1 (mTORC1) being the key regulator of nutrient sensing, which is an important biological hallmark of aging^[1,72]^. Dietary restriction, which is the well-established and likely the most potent intervention to prolong the life span of various organisms, exerts its effects, in part, through the mTORC1 pathway^[72]^. Dietary restriction and reduced intake of methionine and branched chain amino acids by activating the AMP-activated protein kinase (AMPK) targets the mTORC1 for inactivation. The latter plays a central role in longevity in a variety of organisms^[101]^. Moreover, dietary restriction links the mTOR and the FOXO3 pathway through suppression of the insulin/IGF1 signaling and inactivation of PI3K, mTORC2, and AKT, which lead to activation of the FOXO3 transcription factor. Activation of FOXO3 and suppression of the mTORC1 pathway are well established to increase autophagy, improve proteostasis, enhance DNA repairs, improve mitochondrial functions, and reduce oxidative stress, which collectively leads to increased life span as well as healthy aging^[72,101,102]^.

## PERSPECTIVE

Aging is an archetypical and fascinating complex biological process regulated by genetic and environmental factors. Genes and the pathways involved in longevity partly regulate the cellular responses to internal and external stressors, such as metabolic stress, genomic instability, and/or oxidative stress. As discussed earlier, the effect sizes of the genetic variants on aging are projected to follow a gradient, albeit the gradient is expected to be skewed toward those with small effect sizes because of the evolutionary disadvantage of the variants with large effect sizes. As discussed earlier, on one end of the spectrum are the rare monogenic diseases, whereby single nucleotide changes or deletions are sufficient to cause premature aging syndromes. On the other end of the spectrum is the slowly evolving aging phenotype, whereby many genetic variants are operant interactively and stochastically, each imparting a modest effect size. Whereas the genetic basis of the rare monogenic premature aging syndromes is well defined, only a relatively small number of the genetic variants, thus far, have been associated with human life span, longevity, and health span. Moreover, their collective effect sizes have been rather small.

The relative paucity of the genetic variants associated with longevity, as opposed to the discovery of a very large number of genetic variants that are associated with age-related diseases, should not be interpreted to conclude that the genetic factors do not play major roles in life span or longevity. On the contrary, the paucity likely reflects the complexity of the aging process and the modest effect size of each variant, rendering it difficult to discern. Likewise, the small effect size of each variant is reflective of the myriad of the determinants, genetic or otherwise, involved in longevity, i.e., diluting the effect size of each determinant.

The relevance of several genes that have been associated with human longevity in the GWAS to the biological hallmarks of aging has not been clear. These findings should be considered provisional, requiring replication in independent study populations and mechanistic explanations. Alternatively, the GWAS findings might elucidate novel functions of the new genes or uncover new mechanisms pertaining to human longevity. Finally, the genetic factors that influence the responses of the cardiovascular system to stress, including external stress, such as lifestyle and exercise, as well as those affecting the inter-organ interactions and the immuno-metabolic pathways, would be expected to influence longevity and health span.

The impact of the genes on longevity is best illustrated in simpler model organisms, such as *C. elegans* or *Drosophila*, whereby genetic interventions could increase the life span by 4- to 6-fold^[103,104]^. As stated earlier, this review is focused on human molecular genetic studies, and the valuable genetic discoveries made in model organisms are not discussed. Overall, it is fair to surmise that a very large number of genetic variants, albeit each with small effect size, are operant in longevity and have not been identified yet. Therefore, to elucidate the genetic basis of human longevity, innovative approaches would be necessary.

The modest effect size of a genetic variant, however, does not detract from the potential clinical utilities of the findings, as the discovered variants simply point to the involved pathways, which could be the target for interventions. The latter point is best illustrated in the case of the genetic variants in the *LDLR* gene, which impart small effect sizes on the plasma levels of LDL-C, and yet the encoded protein, i.e., LDL-C receptors, is a highly effective therapeutic target for lowering the plasma LDL-C levels^[105–107]^.

Mechanistically, genetic studies have pointed to the involvement of several key events, notably DNA damage, particularly in the progeria/progeroid syndromes and activities of the insulin/IGF1/mTOR/FOXO pathway in longevity. The DNA damage and the cellular responses to it, which includes inflammation, seem to be broadly applicable to aging, yet the evidence for the involvement of the genetic variants in the genes involved in the DDR pathway in longevity, short of progeria/progeroid syndrome, is scant. Whereas inflammation seems to be a ubiquitous phenotypic feature of aging, it is the consequence of several main events and per se is not considered a hallmark of aging as originally defined^[1]^. Likewise, evidence for the commonality of involvement of insulin/IGF1/mTOR pathway in longevity is strong, despite the paucity of the associated genetic variants. The pathway seems to serve as the main mechanism for the beneficial effects of dietary restriction in aging.

A considerable portion, if not the main fraction, of the genes that have been associated with longevity are also associated with age-related phenotypes, such as CAD, systemic arterial hypertension, and diabetes. Naturally, one might also consider such variants as determinants of longevity and the genetics of cardiovascular aging at the core of human longevity. This concept also applies to the genetics of premature aging syndromes, whereby cardiovascular involvement is the main cause of death. The latter set of discoveries, i.e., elucidating the genetic basis of rare premature aging syndromes, provide the most compelling insights into the mechanisms involved in premature cardiovascular aging, which might also pertain to the mechanisms of longevity^[108]^.

The challenges encountered in deciphering the genetic basis of longevity should not distract from the significance of the findings, as the main strength of the genetic discoveries, whether rare forms of premature aging or longevity, pertains to the elucidation of the molecular mechanisms and identification of the pathways that are involved. It is the latter discoveries but not the individual genetic variants that are expected to serve as a target of interventions for a healthy life span.

## Figures and Tables

**Table 1.** Biological hallmarks of aging

	Hallmark^[1]^	Phenotypic description

**Molecular hallmarks**	Genomic instability	An increase in single and double-stranded DNA breaks, the subsequent release of the nuclear (and mitochondrial) DNA into the cytoplasm, activation of the DNA damage response pathway, and inflammation
	Telomere attrition	Human telomeres comprise a repetitive TTAGGG sequence that shortens over time, which triggers senescence
	Epigenetic changes	CpG methylation, histone modifications, and chromatin remodeling are common features of aging
	Impaired proteostasis	Altered expression, folding, trafficking, and degradation of proteins upon aging
	Impaired nutrient sensing	Impaired insulin/IGF1/mTOR signaling pathways lead to suppression of the FOXO transcription factors
**Cellular hallmarks**	Senescence	Loss of replicative function leading to expression of the senescence-associated secretory phenotype
	Mitochondrial dysfunction	The decline in mitochondrial function leads to reduced oxidative phosphorylation, increased oxidative stress, apoptosis, and impaired autophagy
	Impaired cell-cell cross talks	Impaired molecular interactions among cellular constituents of an organ, such as myocytes, endothelial cells, and fibroblast
	Stem cell exhaustion	An imbalance between quiescence and proliferation leads to depletion of the stem cells

IGF1: Insulin-like growth factor 1; mTOR: mechanistic target of rapamycin; FOXO: the forkhead box O.

## Data Availability

Not applicable.
